# Pictorial depth cues elicit the perception of tridimensionality in dogs

**DOI:** 10.1007/s10071-024-01887-1

**Published:** 2024-07-22

**Authors:** Anna Broseghini, Markus Stasek, Miina Lõoke, Cécile Guérineau, Lieta Marinelli, Paolo Mongillo

**Affiliations:** Department of Comparative Biomedicine and Food Science, Università degli Studi di Padova, Viale dell’Università 16, Legnaro, PD 35020 Italy

**Keywords:** Vision, Depth perception, Tridimensionality, Perspective, Violation of expectation, Monocular cues, Dogs

## Abstract

The perception of tridimensionality is elicited by binocular disparity, motion parallax, and monocular or pictorial cues. The perception of tridimensionality arising from pictorial cues has been investigated in several non-human animal species. Although dogs can use and discriminate bidimensional images, to date there is no evidence of dogs’ ability to perceive tridimensionality in pictures and/or through pictorial cues. The aim of the present study was to assess the perception of tridimensionality in dogs elicited by two pictorial cues: linear perspective and shading. Thirty-two dogs were presented with a tridimensional stimulus (i.e., a ball) rolling onto a planar surface until eventually falling into a hole (control condition) or until reaching and rolling over an illusory hole (test condition). The illusory hole corresponded to the bidimensional pictorial representation of the real hole, in which the pictorial cues of shading and linear perspective created the impression of tridimensionality. In a violation of expectation paradigm, dogs showed a longer looking time at the scene in which the unexpected situation of a ball rolling over an illusory hole occurred. The surprise reaction observed in the test condition suggests that the pictorial cues of shading and linear perspective in the bidimensional image of the hole were able to elicit the perception of tridimensionality in dogs.

## Introduction

Visual perception describes the processing of retinal information through the brain in order to interpret physical properties of the world and act upon them (Haber and Hershenson 1973). The final interpretation of visual stimuli is the result of various factors, like visual acuity, color vision, depth perception and spatial resolution, and determines how animals perceive their surroundings. Despite long-standing research on this topic, our knowledge about animal visual perception is far from complete. This shortage is particularly problematic as most experimental tasks used in non-human animals’ studies rely on visual cues and their interpretation of bi- or tri-dimensional stimuli.

As mentioned above, visual perception of depth and tridimensionality is one among the important and adaptive abilities that requires processing of retinal information. The distance of the surfaces from the observer is lost during the process of representing the image on the retina, which is in fact a bidimensional image (Marr and Nishihara 1978). However, the perception of tridimensionality occurs, at least in humans, due to different mechanisms and key clues. Under natural conditions, an observer will use both binocular cues, motion cues, which will both provide quantitative information, and pictorial cues, to estimate tridimensionality (Kim et al. 2016). A well-known mechanism involved in the perception of tridimensionality is binocular vision, whereby the partially overlapping visual fields of the two eyes generate the so-called binocular disparity, i.e., the relative lateral displacement of the viewed object in the two retinal images (Cumming and DeAngelis 2001). The integration of the two images resulting from binocular disparity contributes to create the perception of tridimensionality. Another contribution to the perception of tridimensionality is given by motion parallax, a motion cue which owes to the different perceived velocity of displacement of objects that are at different distances from the observer. Due to this mechanism, objects moving at a constant speed appear to move faster when closer to the observer than objects that are further (Rogers and Graham 1979). Finally, monocular cues, also known as pictorial information, also participate in generating the perception of tridimensionality and are the only ones that can be used when processing bidimensional static stimuli. They include linear perspective, when parallel lines seem to converge in the distance; texture gradient, i.e., the texture of a surface becoming less distinct receding into distance; familiar size, when familiarity with the actual size of an object allows estimation of its distance based on its perceived size; relative size, when the perceived sizes of two objects known to have the same size are compared to estimate their relative distance from the observer; interposition, when distance of objects are inferred by one object partially occluding another; shading, which refers to the change in light reflected from the surface due to the orientation of the surface with respect to the light source; height in the visual field, when objects that appear higher in the visual field are perceived as more distant than objects lower in the visual field; aerial perspective, which refers to the blurring and desaturation of distant objects due to light dispersion by the atmosphere (Palmer 1999). In humans the contribution of these different mechanisms and cues in the perception of tridimensionality is quite well known. Different studies using a real object placed in natural settings found that binocular shape estimation was more veridical than monocular one (Frisby et al. 1996; Loomis et al. 2002; Allison et al. 2009). In the absence of monocular pictorial cues, depth could still be perceived by binocular disparity (Julesz 1964). However, looking at a natural scene with only one eye does not change much about the apparent depth, suggesting that the role of binocular disparity might be overestimated in the perception of depth (Gibson 1950). Moreover, in the presence of a planar surface (i.e. a bidimensional stimulus) with contradictory monocular and binocular information, the interpretation of tridimensionality was driven more by monocular cues rather than binocular ones (Stevens and Brookes 1988). It thus appears that the contribution of binocular and monocular information to the perception of tridimensionality in humans varies according to the visual stimulus (real tridimensional stimuli vs. bidimensional representations of tridimensionality) and available information.

Perception of tridimensionality driven by pictorial cues was also studied in several non-human animal species. The first clear demonstration of animals perceiving bidimensional representations of tridimensionality as real was obtained in baboons. Baboons grabbed and tried to eat the pictorial representation of a banana, suggesting that they did not perceive the photograph as a representation, but as a real banana (Parron et al. 2008). The manipulation of specific pictorial cues was also functional for understanding whether and how these cues are perceived by different species and their relative contribution to the perception of tridimensionality. The pioneering work of Walk and Gibson (1961) studied the relative contribution of texture density to the perception of depth in several species, proving that its importance can vary among species. However, the role of texture density was also demonstrated in species such as cuttlefish (Josef et al. 2014), suggesting that the use of such pictorial cues for the perception of tridimensionality is phylogenetically conserved. Furthermore, baboons have been found to be susceptible to the corridor illusion, which causes equally sized pictures to appear larger at the end of a corridor than at the beginning (Barbet and Fagot 2002). The authors concluded that the subjects were able to infer depth using gradient and perspective cues. The Ponzo illusion also relies on monocular pictorial cues, as linear perspective is created by the two converging lines (Gregory 1963; but see Pressey and Epp 1992 for an alternative explanation). Susceptibility to such an illusion has been proven in several animal species (Bayne and Davis 1983; Fujita et al. 1991; Fujita 1996, 1997; Timney and Keil 1996; Nakagawa 2002).

The extent to which dogs perceive depth and tridimensionality in nature is reflected by their great ability to follow and catch fast-moving objects and overcome obstacles (Miller and Murphy 1995). However, few investigations have been made into dog tridimensionality perception. Binocular vision is possessed by dogs, with a degree of binocular overlap for the average dog likely ranging between 30° to 60° (human binocular overlap is of approximately 140°) (Miller and Murphy 1995). However, studies of retinal ganglion cell topography (Peichl 1992) have hypothesized that depth perception in dogs might be impaired, due to a lack of alpha ganglion cells in the peripheral 15° of the right and left portions of the area of binocular overlap. This means that the area of the retina designated for high-quality depth perception might be smaller than what estimated by binocular overlapping. There is also limited evidence for dog’s ability to perceive linear perspective cues related to depth perception since dogs were found to be not susceptible to the Ponzo illusion (Byosiere et al. 2018). Puppies subjected to the experimental condition of the visual cliff displayed proper depth perception (Walk and Gibson 1961), even if the relative contribution of pictorial cues (i.e. texture density) was not investigated. Although dogs have similar abilities to humans in the perception of motion (Kanizsár et al. 2017, 2018), to the best of our knowledge, no studies have investigated the role of motion cues for depth perception in dogs. On the other hand, numerous studies have demonstrated the ability of dogs to use or discriminate bidimensional images, suggesting, albeit indirectly, their ability to perceive their tridimensionality (and therefore to use pictorial cues) (Albuquerque et al. 2016; Autier-Dérian et al. 2013; Eatherington et al. 2020; Huber et al. 2013; Müller et al. 2015).

The aim of the present study is to shed light on dogs’ perception of tridimensionality. Knowing how dogs perceive pictorial cues is of relevance for both applied and scientific contexts. As mentioned above, visual illusions were used to study tridimensional perception in animals. In fact, the appropriate manipulation of pictorial cues allows to create the illusory perception of depth and tridimensionality exactly as it happens in paintings. Visual illusions were initially studied mostly in human infants or adults to understand perceptual biases affecting our interpretation of color, size or motion (Kanazawa et al. 2013; Schlaffke et al. 2015; Agrillo et al. 2019), but susceptibility to visual illusions has been found across other taxa. Accordingly, it is assumed that at least part of the mechanisms leading to the misinterpretation of the physical reality may be shared by humans and non-human animals (Feng et al. 2017). Taking advantage of this possibility, we exposed each dog to two experimental conditions. In one condition, a ball was rolling onto a planar surface, until falling into a real hole (control condition); in the other condition, the ball rolled onto the same surface until reaching a bidimensional pictorial representation of a hole (henceforth referred to as Illusory hole) and therefore rolling over the hole itself (test condition). Using a violation of expectation paradigm, an experimental approach that has been previously and successfully used in dogs (Mongillo et al. 2021), we hypothesized that if dogs perceived the pictorial cues of the Illusory hole a surprised reaction (i.e. longer looking time at the area where the unexpected situation occurred), would occur, because of the ball expectingly rolling over the illusory hole.

## Methods

### Subjects

For this study, forty companion dogs were initially recruited, along with their caregivers who participated in the experiment on a voluntary basis. The caregivers were either students at University of Padua or recruited via the Laboratory of Applied Ethology database of volunteers. The selection criteria were for the dogs to have a good health condition, absence of vision problems, ease of adaptation and interaction in a new environment and a height at the eye level between 35 and 100 cm in sitting position. The final sample consisted of 32 dogs (12 males, 20 females; average age ± SD = 4.8 ± 2.7 years; 5 Mixed breed, 4 French Bulldogs, 2 Bernese Mountain dogs, 2 Siberian Husky, 2 Irish Red Setter, 2 Labrador, 1 boxer, 1 Eurasier, 1 Podenco Canario, 1 Dachshund, 1 Maltese, 1 Golden Retriever, 1 English Cocker Spaniel, 1 Border Collie, 1 Newfundland, 1 White Swiss Shepherd Dog, 1 Shiba Inu, 1 Samoyed, 1 Cavalier King Charles Spaniel, 1 Lagorai Shepherd, 1 Pug). The remaining eight subjects were excluded for technical problems with the video recording, presence of external distractions, or substantial lack of attention of the dog on the ball at the start of the trial (see below). As the experimental design relied on obtaining valid data from the two conditions for any given subject, even if only one of the two trials was compromised, both trials were excluded from analysis for that dog.

### Experimental setting

**Fig. 1:**
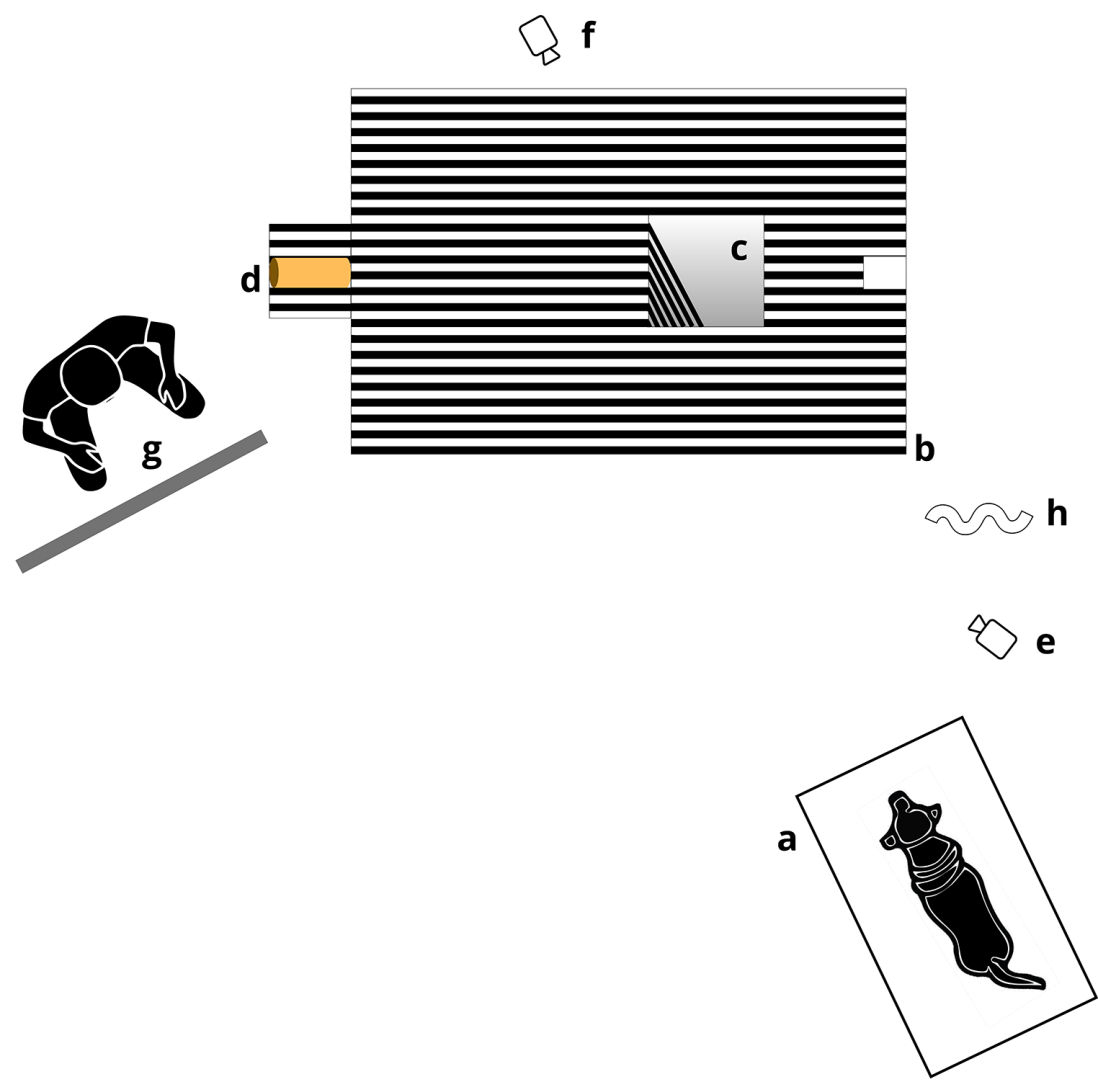
Representation of the experimental setting, illustrating the position of the dog on the platform (**a**), the experimental apparatus (**b**), the illusory/real hole (**c**), the paper tube into which the ball is dropped (**d**), the camera focusing on the apparatus (**e**), the camera focusing on the dog’s eyes (**f**), the experimenter who placed the ball into the tube, hidden behind the opaque panel (**g**), the white curtain (when closed) to hide the panel (**h**). The caregiver, sitting on the platform holding the dog, the second experimenter, who gives instructions to the caregiver, the ceiling camera focusing on the dog’s head are not depicted. The elements of the represented figure are not scaled

The test was carried out in an experimental room (4.7 × 5.8 m), which was protected from outside noise as much as possible. The experimental setup is presented in Fig. 1. A blanket-covered platform was placed as a sitting place for the dog and the caregiver during the experiment. The platform was orientated towards the experimental apparatus, and in particular its axis was aligned with the direction of the lines of the illusory hole image. The platform was composed of a variable number of rectangular polyurethan foam elements (60 × 125 × 5 cm), which were progressively added to obtain the height of the dog’s eyes when sitting approximately 100 cm above the floor.

**Fig. 2:**
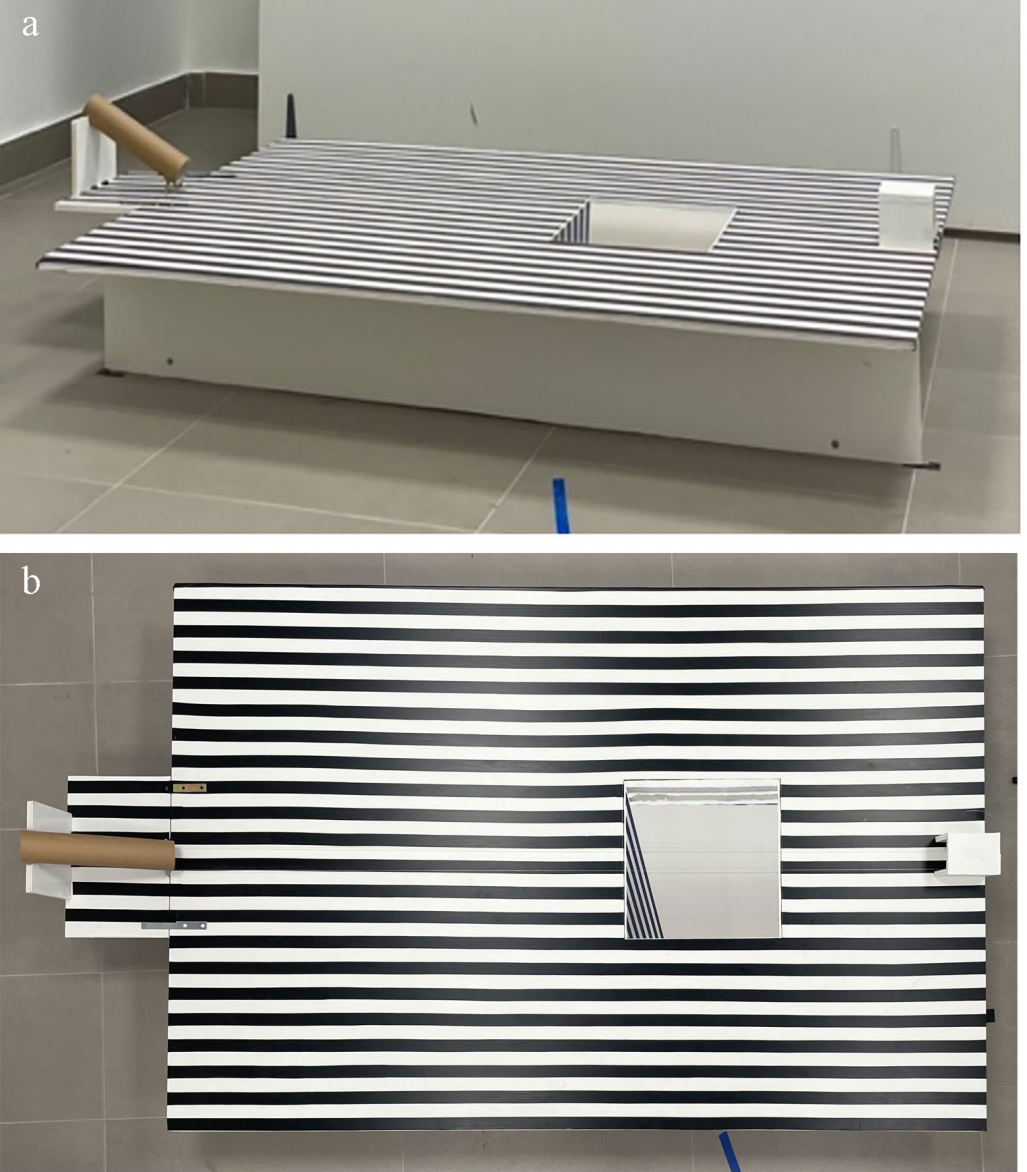
Pictures of the apparatus in the experimental conditions “Illusory hole” viewed from the dog’s point of view (on the left) and from above (on the right). In this condition, a plastic board (30 × 30 × 1 cm) depicting a black and white striped cliff was placed on top of the real hole to create the illusion of a hole, which was perceivable at best from the dog’s point of view

The experimental apparatus was placed 240 cm away from the platform and consisted of a plastic board (152 × 102 × 1 cm), supported by plastic feet to be 25 cm above the floor. The upper surface of the board was covered with alternating black and white stripes (width 2.5 cm). A hole (30 × 30 cm) was cut in the board, starting at 85 cm from the left end of the apparatus and at the middle of its width. The lateral sides of the hole were made of plastic walls; the walls parallel to the long axis of the apparatus were covered with alternating black and white stripes, in continuity with those on the upper surface of the board. The bottom of the hole was closed with a net invisible to the dog to prevent the ball from bouncing off the floor when it fell into the hole. The hole could be left open or closed with a panel to create the experimental conditions “Real hole” and “Illusory hole”, respectively. The panel used to create the “Illusory hole” condition consisted of the printing of the real hole’s photograph, taken from the perspective of the dog. Therefore, this panel contained the black and white stripes, as well as shadows, as bidimensional pictorial cues of the hole (Fig. 2). On the left side of the board, a paper tube (diameter 5 cm) was attached with an inclination of 30°, through which the ball (diameter 4 cm) was dropped during the test, so that it acquired the speed necessary to cross the entire board. At the opposite end of the board a small box (15 × 9 × 8 cm) was placed to collect the ball, in the case the hole was closed, i.e., in the illusory hole condition. To ensure that the ball ran straight towards the hole or the small box, two transparent fishing lines were used. The lines ran from either side of the paper tube to the box at the other end of the board, slightly raised from the board surface, thereby creating a physical lane within which the ball was kept during its run. Next to the apparatus, at the left end of the paper tube, an experimenter was hidden behind an opaque panel; her role was to place the ball in the tube. This panel had a small slit at a height of 90 cm, which was covered by a dense net of the same color as the panel, allowing the experimenter to see the dog’s head not being seen by the dog. The areas behind the apparatus and next to the platform were also delimited by opaque panels to create a homogeneous background. In front of the apparatus, a white curtain was placed to hide the apparatus until the start of the experiment. Blue tape stripes were placed on the floor of the experimental room to delimit different areas toward which the dog could be orientated to facilitate subsequent data collection.

Three video cameras were used for the recording of the experiment. A CCTV camera was mounted on the ceiling to monitor the orientation of the dog’s head, one was placed on a tripod orientated towards the apparatus (Sanyo Xacti HD Dual Camera, Tokyo, Japan) and the third (Canon XA20, Tokyo, Japan) was orientated toward the dog’s face through a hole in one of the background panels. This last camera was recording the dog’s eye orientation, to confirm the agreement of the orientation of the eyes with the orientation of the head.

### Procedure

Before starting the experiment, the distance between the dog’s eyes and the floor was measured to adjust the height of the sitting platform. Immediately afterward, the owner and the dog entered the testing room and the owner was instructed about the procedure. The owner was asked to sit on the platform, position the dog between the legs and gently hold the dog throughout the experiment, stay still, do not interact with the dog and look down straight in order not to influence the dogs’ behavior during the test. Once everyone was in the right position, the experimenter opened the curtain to reveal the apparatus and hid behind the panel on the left side of the apparatus. A second experimenter, who was already hiding behind the panel next to the apparatus, sticked out the hand holding the ball to draw the dog’s attention. The intensity of the attention grabber increased until the dog was clearly attracted to the ball, from the simple movement of the hand to the movement of the hand associated with calling the dog’s name, and finally the call of the dog’s name with an excited voice and rapid hand movement. If after 10 s the dog was still not attracted and focused on the ball, it was excluded from the testing. As soon as the dog was attentive, the experimenter released the ball into the paper tube. Thirty seconds after the ball fell either into the hole or into the box at the end of the apparatus, the trial ended, and the owner and the dog left the room.

Each dog underwent two trials as described above, one in the “Real hole” condition and one in the “Illusory hole” condition. The order of presentation of the two conditions was counterbalanced across the sample. Between the first and second condition, the caregiver was asked to wait with the dog in a separate room for five minutes.

### Data collection and analysis

Data collection of dog head and eye orientation was conducted with Observer XT software (version 12.5, Noldus, Groeningen, The Netherlands), with a continuous sampling technique. Based on the tape lines on the floor, the orientation of the dogs’ head was coded as toward the ‘beginning of the apparatus’ (from the panel hiding the experimenter to the beginning of the hole), the ‘end of the apparatus’ (from the hole to the end of the apparatus) and ‘elsewhere’ (anywhere else in the room). The coders only had access to the videos that pointed towards the dogs’ head, so they were blinded to the experimental condition. The head orientation data was collected from the moment the dog was attentive to the ball until 30 seconds after the ball fully disappeared. To assess interobserver reliability, 20% of the videos were coded by a second person. Interobserver reliability was considered very good for all data collected (ICC ‘beginning of the apparatus’ = 0.96; ICC ‘end of the apparatus’ = 0.92; ICC ‘looking elsewhere’ = 0.82). After the dog’s head orientation was coded, the coders collected data on the timing of the ball’s appearance and disappearance and of the start and end of trial.

To assess whether dog’s attention was equally attracted by rolling ball in both conditions, paired t tests were applied to compare dog’s looking time at the beginning of the apparatus between conditions, starting from the moment the ball first appeared outside the tube to the moment the ball reached the beginning of the hole.

According to the violation of expectation paradigm, if dogs were sensitive to the pictorial representation of the hole, they should be surprised by the fact that the ball kept rolling without falling into the hole. In this paradigm, the surprise is represented by an increased attention towards the area where the phenomenon has occurred compared to a control condition (Winters et al. 2015). In our procedure, the phenomenon that could possibly violate the expectation of the dog occurred in the ‘end of the apparatus’ area. Therefore, we used a Generalized Estimating Equation (GEE) model to analyze the duration of the dog’s orientation towards the ‘end of the apparatus’ during the 30 s after the ball fully disappeared, under the two experimental conditions. The looking time at the end of the apparatus was included as a dependent variable in the model. The experimental condition, trial order and interaction between these two factors were included as independent variables. The identity of the dog was included as a random effect to account for repeated measurements in the same subject. The same model was also used to analyze the duration of the dog’s orientation towards the other areas including looking at ‘beginning of apparatus’ and looking ‘elsewhere’ as dependent variables.

All statistical analysis were performed with SPSS (v. 28, IBM, Armonk, NY). The level of statistical significance was set at 0.05.

## Results

The trials in the “Illusory hole” condition, from the moment the ball appeared to 30 s after the ball disappeared, lasted for 31.6 ± 0.2 s. In the “Real hole” condition, the trials lasted for 31.0 ± 0.1 s.

When visible, the ball almost completely attracted the dog’s attention. The subject’s heads were oriented to the beginning of the apparatus for 91.2 ± 13.9% of the 0,9 s time interval between the release of the ball and the moment in which it reached the hole. Dogs’ looking time at the beginning of the apparatus during this time interval did not significantly differ between the two experimental conditions (Illusory hole condition = 0,80 ± 0,15 s; Real hole condition = 0,83 ± 0,09 s; t = − 0.881, df = 31, *p* = 0.385, Cohen’s d = -0.156). In the “Illusory hole” condition, the looking time at the end of the apparatus from the instant the ball reached the illusory hole until it fell into the box at the end of the apparatus was also high (72.9 ± 32.4%).

**Fig. 3:**
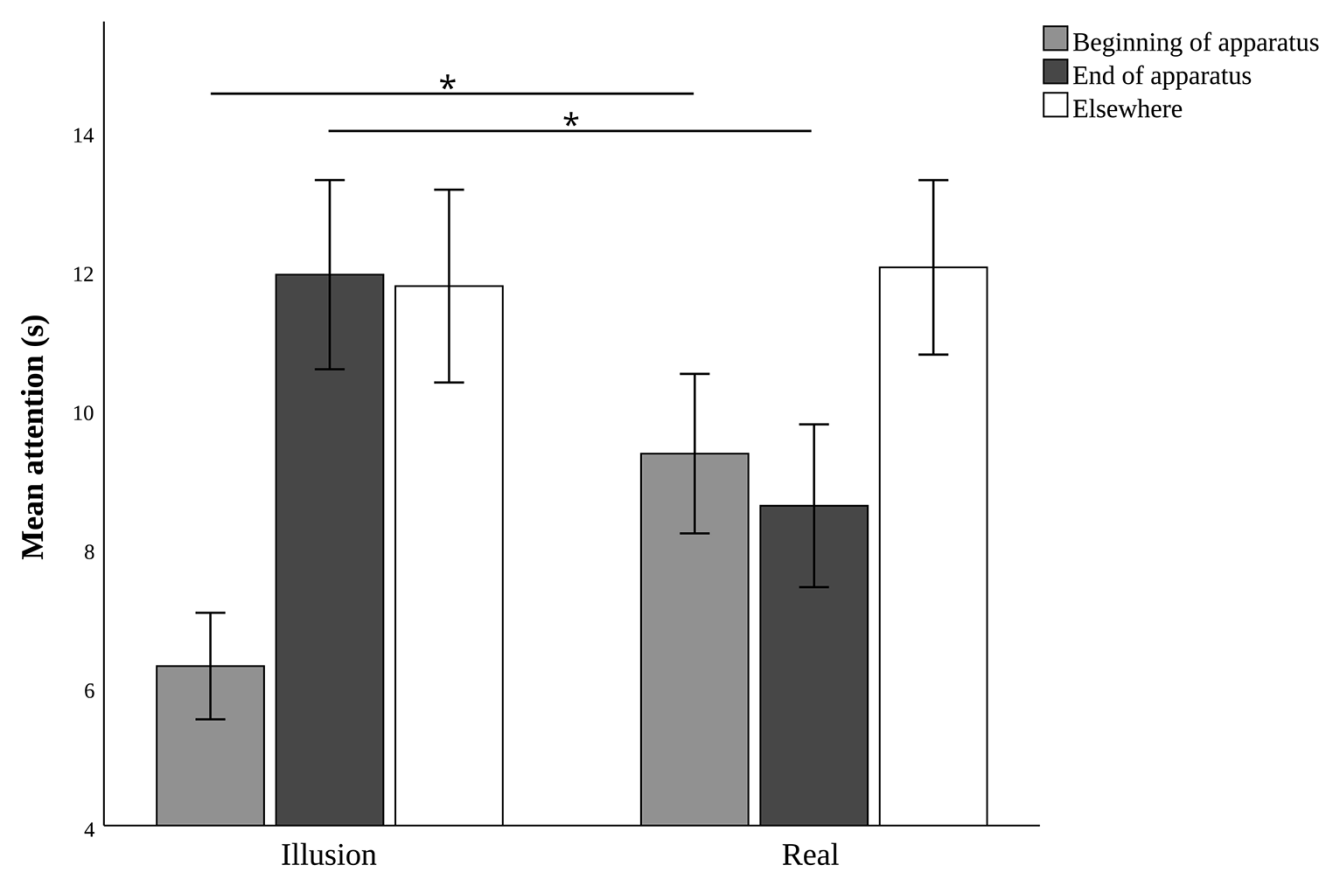
Mean attention (± SE) paid by dogs to different parts of the experimental setting, in the Illusion and Real conditions, during the 30 s after the ball disappeared

During the 30 s time interval after the ball disappeared, the amount of attention paid by the dogs to the different parts of the apparatus under both experimental conditions is reported in Fig. 3. The GEE model revealed that the condition had a significant effect on the subject’s looking time at the end of the apparatus in the 30 s after the ball fully disappeared (Wald Chi-square = 6.440, *p* = 0.011, Cramer’s v = 0.984), being significantly higher in the “Illusory hole” condition (estimated mean ± std. error = 11.88 ± 1.33 s) than in the “Real hole” condition (estimated mean ± std. error = 8.58 ± 1.15 s). The order of condition did not affect the looking time at the end of the apparatus (Wald Chi-square = 0.194, *p* = 0.659), nor did the interaction between these variables (Wald Chi-square = 0.363, *p* = 0.547). Moreover, the GEE model showed that the condition had a significant effect on the subject’s looking time at the beginning of the apparatus in the 30 s after the ball fully disappeared (Wald Chi-square = 7.825, *p* = 0.005, Cramer’s v = 1), being significantly higher in the “Real hole” condition (estimated mean ± std. error = 9.29 ± 1.10) than in the “Illusory hole” condition (estimated mean ± std. error = 6.26 ± 0.74). The order of condition did not affect the looking time at the end of the apparatus (Wald Chi-square = 0.163, *p* = 0.686), nor did the interaction between these variables (Wald Chi-square = 1.066, *p* = 0.302). Finally, the dog’s looking time elsewhere in the 30 s after the ball fully disappeared was not different between the two conditions (Wald Chi-square = 0.062, *p* = 0.803), the order of condition (Wald Chi-square = 0.016, *p* = 0.898) and the interaction between these variables (Wald Chi-square = 1.509, *p* = 0.219).

## Discussion

The present study assessed dogs’ reaction to a ball rolling over an illusory hole, as an experimental condition to evaluate their use of pictorial cues in perceiving tridimensionality.

The dogs showed great interest in the stimulus rolling along the apparatus as the ball attracted the dogs’ attention in a sustained manner throughout the trial, regardless of the experimental condition. This is not surprising since, for most pet dogs, balls are relevant objects, associated with pleasant experiences such as play and interaction with the owner (Abdai et al. 2017). Moreover, motion detection is a crucial adaptive ability of dogs (Kanizsár et al. 2017), who are more sensitive to moving objects than stationary ones (Miller and Murphy 1995). These results support the appropriateness of the experimental setting and the procedure used in the present study.

Our results also indicate that dogs perceived the illusory hole as a real one, since they showed a surprise reaction (i.e., longer looking time in the area where the unexpected phenomenon occurred) after witnessing the ball rolling over the illusory hole compared to the control condition. The absence of a significant order effect excludes that the result was due to an expectation generated by exposure to the real hole condition. In fact, in the case the first presentation was the real hole condition, the surprise reaction observed in the following illusory hole condition might be due to the dog’s expectation of the ball falling into the hole as in the previous trial. However, if this was the case, when the first presentation was the illusory hole condition, the dogs had no expectations and should not present any surprised reactions. Our results do not support this explanation since the presentation order had no effect on dogs’ looking time and the dogs showed surprised reaction in the illusory hole condition regardless of its presentation order. Thus, the best explanation for the dogs’ looking time after the stimulus disappeared is that the dogs perceived the illusory hole as real regardless of the hole being a bidimensional representation. The analysis also revealed that the dogs looked longer at the area ‘beginning of apparatus’ in the control condition than in the test condition. Therefore, in the absence of an unexpected event, the dogs’ attention is directed to the area of the experimental setting where some activities occurred. This is not surprising since the ‘beginning of the apparatus’ is the area where the dog’s attention was initially captured and from where the ball starts travelling, making it interesting for the dogs.

The ability of dogs to perceive tridimensionality in the present study arose from the manipulation of specific pictorial cues of a bidimensional image. Previous studies already showed that dogs respond appropriately to bidimensional stimuli depicting tridimensional items. Pictures have been used to test different dogs’ abilities, such as recognition and discrimination of species, human emotions, and faces (Autier-Dérian et al. 2013; Huber et al. 2013; Müller et al. 2015; Albuquerque et al. 2016; Eatherington et al. 2020). However, the stimuli used in the above-mentioned studies were much more complex than the ones we used, providing dogs with numerous characteristics and features of the bidimensional stimuli useful for recognition. Moreover, perceived tridimensionality by the dog was not the aim of these studies, thus not allowing to clarify if and how dogs can perceive tridimensionality in bidimensional representations, and which characteristics are relevant for the processing of pictorial stimuli.

In our study, the depth cues available in the experimental scene were monocular pictorial cues represented by linear perspective and shading, and binocular cues, i.e., binocular disparity, as dogs were looking at the scene with both eyes. However, binocular cues are not helpful when observing bidimensional image, as the two eyes, presented with a flat surface picture, are not receiving two different perspectives of the same scene. Accordingly, the present results support the fact that binocular disparity did not play a significant role in the perception of tridimensionality of the hole. Indeed, if binocular disparity had played a relevant role in the perception of tridimensionality, the dogs should not have been surprised in the illusory hole condition, as the absence of binocular cues in the pictorial representation of the hole should have promoted the perception of the hole as a planar surface. Nevertheless, the pictorial representation of a hole presented in our experiment was embedded in a real tridimensional apparatus, just as the stimulus/ball was a tridimensional object. Hence, we cannot completely exclude that binocular cues played some role in favoring the tridimensionality perception of the hole by the dogs.

As mentioned before, the monocular pictorial cues were specifically designed to make the pictorial representation resemble the tridimensionality of the real hole. To reach this aim, in the pictorial representation the vertical side of the hole presented parallel lines oriented along the theoretical vertical axis, but in fact directed towards the viewer, and a gradual decrease in brightness (or a gradual increase in shade) as the illusory hole deepened. The study of pictorial depth cues in non-human animal is quite widespread (e.g., chicks: Regolin and Vallortigara 1995; Hershberger 1970; macaques: Gunderson et al. 1993; Hanazawa and Komatsu 2001; chimpanzee: Imura and Tomonaga 2003; cuttlefish: Josef et al. 2014; horses: Timney and Keil 1996). Among these, some studies specifically investigated the pictorial cues of the present research. In line with our results, pigeons trained to distinguish pictures of non-manipulated objects from images of objects where shading cues and perspective cues were manipulated were able to perceive and use both types of cues (Reid and Spetch 1998). Through a preferential reaching task, chimpanzee infants were shown to be able to perceive shading information of pictorial depth cues (Imura and Tomonaga 2003). Moreover, paradigms, such as the Corridor illusion (Barbet and Fagot 2007) or the Ponzo illusion (Gregory 1963), have also been used to assess the perception of linear perspective in animals. As mentioned in the Introduction, dogs are not susceptible to the Ponzo illusion at a group level (Byosiere et al. 2018), which questions their ability to use perspective cues. However, it should be highlighted that the biased perception assessed in such illusion involves not only the use of linear perspective cues but also the estimation of item size based on linear perspective rules. Thus, the susceptibility of dogs to linear perspective cues does not directly conflict with the results of Byosiere and colleagues (2018). Presented with the Ponzo illusion, dogs could indeed have failed to use perspective as a size cue, while still being able to use perspective cues as information to perceive depth. Moreover, differences in the experimental setting between the two studies should be highlighted, since the use of a tridimensional apparatus and an attractive stimulus such as the ball in our study may have elicited higher levels of attention from dogs and a more natural response. Finally, it is also possible that additional pictorial cues (i.e. shading) are needed for dogs’ successful use of perspective cues in the perception of depth and tridimensionality.

The fact that the ball did not fall into the illusory hole is not the only possible explanation for dogs increased attention in the experimental condition. Although dogs did not move during the experiment, their heads were not restrained allowing for potential small lateral head movements. This could have sufficiently altered the viewpoint, so that the orientation of the lines of the illusory hole would no longer be in accordance with the correct perspective, revealing the two-dimensional nature, or at least the oddity of the image. In turn this, rather than the rolling of the ball above the hole, might have attracted dogs’ attention after the exposure. However, the amount of attention paid by dogs to the rolling ball, along with the fact that head movements were never actually seen, would suggest against this explanation. More importantly, the explanation would still be an indication that dogs perceived the illusory hole as a three-dimensional structure, reacting surprised to the misalignment of the lines with their theoretical vertical orientation. Yet, this consideration raises the question of the relative contribution of shading and linear perspective in eliciting depth perception in dogs, when observing bidimensional representations of tridimensional objects. Comparative studies hypothesized that the importance of different pictorial cues to three-dimensional perception might have been shaped by evolutionary constrains, linked to the living environment of each species (Imura and Tomonaga 2003). In line with this hypothesis, the importance of perspective cues may be greater in species that live in large, open spaces, while shading may be more important for animals that live in visually crowded environments (e.g. a forest). Since dogs and wolves are not strictly confined to environments with any of these characteristics, it is difficult to make predictions on which of the two features, if any, would be prevalent in these species. Therefore, the current experiment indicates that monocular pictorial cues elicit the perception of three dimensionality in dogs and prompts for more studies to address the individual importance of shading and linear perspective to such visual process.

Finally, present results do not negate that non-pictorial cues, i.e. binocular disparity, might also be relevant for perception of three-dimensionality in dogs. The basic existence of such mechanism has been demonstrated even in non-visual specialist mammals, such as mice (Boone et al. 2021). However, it was also suggested that regulatory mechanisms of binocular disparity are much different between mice and primates (Samonds et al. 2019), but little is known about other species. Extending our knowledge of the mechanisms underlying three-dimensional perception and stereopsis in dogs would therefore be of relevance from a comparative standpoint.

## Data Availability

Data are publicly available in the data repository of the University of Padua, at https://researchdata.cab.unipd.it/id/eprint/1205. Last access 21/02/24.
